# Physical Activity in Adolescent Females with Type 1 Diabetes

**DOI:** 10.1155/2010/328318

**Published:** 2010-06-24

**Authors:** Bahareh Schweiger, Georgeanna Klingensmith, Janet K. Snell-Bergeon

**Affiliations:** Barbara Davis Center for Childhood Diabetes, The Children's Hospital Aurora, University of Colorado Denver, CO 80045, USA

## Abstract

*Objective*. We sought to identify amount of physical activity and relationship of physical activity to glycemic control among adolescent females 11 to 19 years of age with type 1 diabetes mellitus (T1DM). We also sought to evaluate associations of age and ethnicity with physical activity levels. *Research Design and Methods*. Adolescent females ages 11–19 years (*n* = 203) were recruited during their outpatient diabetes appointment. Physical activity was obtained by self-report and was categorized as the number of days subjects had accumulated 60 minutes of moderate-to-vigorous physical activity during the past 7 days and for a typical week. *Results*. Girls reported being physically active for at least 60 minutes per day on 2.7 ± 2.3 days in the last week, and on 3.1 ± 2.2 days in a typical week. A greater number of physically active days in a typical week were associated with lower A1c (*P* = .049) in linear regression analysis. *Conclusion*. Adolescent females with T1DM report exercising for at least 60 minutes about 3 days per week, which does not meet the international recommendations of 60 minutes of moderate-to-vigorous activity per day. It is particularly important that adolescent girls with T1DM be encouraged to exercise since a greater number of physically active days per week is associated with better glycemic control.

## 1. Introduction

The benefit of physical activity in type 1 diabetes (TIDM) was appreciated well before the more recent improvement and advances in diabetes treatment introduced by the Diabetes Control and Complications Trial (DCCT) [1–3]. Physical exercise in adolescents with TIDM is associated with improved lipid levels and lipoprotein (a) [4]. It has also been found to have a positive effect on blood glucose control by improving insulin sensitivity, stimulating muscle glucose uptake, and subsequently leading to decreased insulin requirements [5, 6]. Other studies have found that not only is there an improvement in insulin sensitivity but that Physical Working Capacity, a measure of aerobic fitness, is also found to be improved with increased energy expenditure in adolescents with TIDM [7]. The long-term benefits of exercise also include a reduced risk of coronary artery disease and stroke [8]. Thus, routine physical exercise is integral in helping to prevent chronic diseases in all children, including those with diabetes.

There are not only physical but also emotional benefits to routine physical exercise [9]. In a study by Wiesinger, 23 otherwise healthy patients with history of TIDM underwent a 4-month exercise study and were found to have both improved metabolic control and also Health Related Quality of Life, a measure of daily functioning and general well being [10]. Other studies have also found that physical activity is associated with a reduction in the symptoms of depression and anxiety [11] and with an improvement in self-esteem [12]. Physical activity is therefore important in helping to foster growth and development during childhood.

However, recent data on the amount of physical activity among adolescent females with TIDM and its association with glycemic control are limited and often conflicting. Some studies have shown that improved A1c is associated with increased physical activity in women [13], but others have not found an association [14–16]. The few studies that have looked at physical activity in adolescent females are concerning in that they have found that there is a tendency toward less physical activity [15–17] and increased prevalence of obesity among adolescent females with TIDM [18]. Thus, it is important to better understand if adolescent females with TIDM are also at increased risk for less physical activity and to better understand the relationship between physical activity and glycemic control.

The aim of the present study is to investigate the relationship of physical activity to glycemic control in large group of adolescent females ages 11 to 19 years of age with TIDM. In addition, we aim to evaluate associations of age and ethnicity with physical activity levels, and to see what proportion of adolescent girls with T1DM are currently meeting the recommended guidelines for exercise.

## 2. Materials and Methods

Subjects were recruited from individuals with T1DM who receive their care at the Barbara Davis Center for Childhood Diabetes. We enrolled females of all ethnicities, defined by the standard 2000 US census questionnaire between the ages of 11 and 19 years of age, and those who had at least one menstrual period were included in the analysis. Subjects with any renal, respiratory, or cardiac disease and any other chronic disease besides TIDM were excluded from the study. Subjects with a preexisting diagnosis of Hashimoto's thyroiditis were included if their initial diagnosis was made by close surveillance and if they had a longstanding history of being clinically and biochemically euthyroid. A total of 234 subjects were enrolled in the study. 15 subjects were excluded because they were 20–24 years of age. Complete data on age at T1DM diagnosis and level of physical activity were available for 203 females with T1DM between the ages of 11 and 19 years of age. Since subjects were randomly recruited during their routine visit, they well represented the adolescent females with TIDM at the BDC. Subjects were randomly approached to partake in the study based on there age and female gender. The study took place between November 2008 and May 2009. Study approval was obtained from the institutional review board at the University of Colorado Denver and participants provided written informed consent and assent, if appropriate, at enrollment.

### 2.1. Data Collection

The clinical diabetes type assigned by the health care provider was obtained from the medical records and categorized as T1DM if the provider assignment was autoimmune TIDM or idiopathic TIDM [19]. Blood pressure, pulse, height, weight, and A1c were measured at the study visit. For measurement of HbA1c the DCA 2000 manufactured by Bayer was used. The reference value for healthy persons is 4–6%. Blood pressure and pulse were measured in the resting sitting position using 4200 Vital Check equipment. Weight was measured in kg using the Detecto scale. Height was in centimeters by use of Holtain Limited Stadiometer. BMI was calculated as kg/m^2^, and BMI *z* score was determined using age and sex specific BMI percentiles from the Centers for Disease Control and Prevention (CDC). Physical examination was performed by a study investigator. Subjects were asked to fill out four short medical history questionnaires. (1) Screening Demographic Questionnaire, (2) Physical Activity Questionnaire, (3) Menstrual Cycle Questionnaire, and (4) Insulin Record Questionnaire. 

The Screening/Demographic Questionnaire asked study participants if they were of Spanish or Hispanic origin or descent. They were also asked which ethnicity they considered themselves to be using the standard 2000 US census questionnaire. In the present study we used a validated questionnaire entitled PACE + (Patient-Centered Assessment and Counseling for Exercise Plus Nutrition) Adolescent Physical Activity Measure survey [20]. Physical activity was obtained by self-report and was categorized as the number of days subjects had accumulated 60 minutes of moderate to vigorous physical activity during the past 7 days and for a typical week and consisted of two questions. Moderate physical activity was defined as “usually makes you breathe hard or feel tired some of the time” and examples were provided: brisk walking, weight lifting, and yard work. Vigorous physical activity was defined as “usually makes you breathe hard or feel tired most of the time” examples were provided: jogging, soccer, and “aggressive” skateboarding. The first question stated “Over the last 7 days on how many days were you physically active for a total of at least 60 minutes per day? The second question stated “Over a typical or usual week, on how many days are you physically active for a total of at lease 60 minutes per day? Both questions had 7 answer choices ranging from 0 days to 7 days per week [20]. 

Age of the first menstrual period and menstrual cycle pattern and length was obtained by self-report using a questionnaire adapted from the National Health and Nutrition Examination Survey reproductive health questionnaire. The Insulin Record Questionnaire included the number of blood glucose checks the patient performs per day, insulin regimen, and doses. Insulin dose was analyzed as average units of insulin used on a typical day per kilogram of weight per day.

### 2.2. Definitions

The 2005 physical activity recommendations by the CDC states that school-age youth should participate daily in 60 minutes or more of moderate to vigorous physical activity that is developmentally appropriate, enjoyable, and involves a variety of activities [21], and the American Diabetes Association (ADA) [22] adheres to the CDC recommendations. The American Academy of Pediatrics (AAP) has similar recommendations and encourages children and adolescents to be physically active for at least 60 minutes per day, which does not need to be acquired in a continuous fashion but rather may be accumulated by using smaller increments. Events should be of moderate intensity and include a wide variety of activities as part of sports, recreation, transportation, chores, work, planned exercise, and school-based PE classes [23].

## 3. Results

Complete data on age at T1DM diagnosis and level of physical activity were available for 203 females with T1DM between the ages of 11 and 19 years. There were 23 subjects with stable, treated hypothyroidism included in the analysis. Characteristics of the study participants are shown in Table 1. The average number of days that subjects reported being physically active for at least 60 minutes per day over the last week was 2.6 ± 2.3 (mean ± SD) days, and for a typical week was 3.1 ± 2.2 days.

Days of physical activity of at least 60 minutes duration in the past week (OR = 0.98 [0.86–1.11], *P* = .73) and typical number of days of physical activity of at least 60 minutes duration (OR = 0.99 [0.85–1.11], *P* = .85) were not associated with irregular periods. However, a greater number of physically active days in a typical week were associated with lower A1c (*P* = .049) in linear regression analysis.

### 3.1. Age

Level of physical activity was also evaluated by two subgroups: 11–15 years and 16–19 years. As shown in Table 2, adolescent females in the 16–19 year age range reported significantly fewer physically active days in a typical week and in the previous week than girls in the 11–15 year age range. In linear regression analysis adjusted for race/ethnicity and Tanner stage, the number of physically active days in a typical week remained significantly lower in the 16–19 year age group (LS mean 2.7) compared to the 11–15 year age group (LS mean 3.9, *P* = .0002).

### 3.2. Race/Ethnicity

Level of physical activity by race/ethnicity is shown in Table 1. Females of Hispanic origin in comparison to non-Hispanic white (NHW) reported fewer physically active days in a typical week (2.3 ± 2.1 versus 3.3 ± 2.1 *P* = .015 and in the last week (1.90 ± 2.0 versus 2.9 ± 2.3 *P* = .031). In multivariate linear regression analysis the typical number of physically active days per week was significantly greater in NHW girls (least square mean ± SE = 3.66 ± 0.28 days) than in Hispanic girls (least square mean ± SE = 2.64 ± 0.46 days, *P* = .014), adjusting for age group and Tanner stage.

### 3.3. ADA Recommendations

Only 10 out of 213 adolescent females (4.7%) met the recommended 60 minutes of physical activity 7 days per week. In a typical week, 35% of adolescent females reported engaging in at least 60 minutes of physical activity 5 or more days per week, while 30% engaged in 1 day or less of physical activity lasting at least 60 minutes. Those with the highest physical activity (5 or more days per week) compared to the least active (1 or fewer days per week) were younger (mean age 15.1 ± 1.8 versus 16.2 ± 2.5 years, *P* = .007), had better glycemic control (mean A1c 8.9 ± 1.7 versus 9.6 ± 2.1, *P* = .026), a lower BMI (22.7 ± 3.7 versus 24.3 ± 3.9, *P* = .019), and lower systolic blood pressure (110 ± 16 versus 116 ± 10, *P* = .012), and diastolic blood pressure (64.3 ± 8.6 versus 68.5 ± 9.8, *P* = .009).

## 4. Discussion

The present study is the largest to date to examine level of physical activity among an unselected group of adolescents and young adults (ages 11 through 24 years) with TIDM studied when they presented for their routine diabetes clinic appointment. The results of this study indicate that adolescent females with T1DM are typically engaged in at least 60 minutes of moderate to vigorous physical activity about 3 days per week, which is similar to results found in other studies [24]. The reported frequency of physical activity in the present study falls short of meeting the guidelines of the AAP [23], the ADA [22], and the CDC [21], which recommend that children engage in at least 60 minutes of physical activity per day.

As expected, we found a correlation between the number of physically active days and A1c, in agreement with findings in some previous studies [13]. This is of important clinical significance since the DCCT [3] and other studies [25] have demonstrated conclusively that improved glycemic control, as assessed by A1c, markedly reduces the development of and progression of microvascular complications of diabetes such as retinopathy, neuropathy, and nephropathy. However, it should also be noted that other studies have not found an association between physical activity and HbA1c [14–16].

We also found that Hispanic females with TIDM were overall less physically active than NHW adolescents with TIDM. These results are similar to other studies that have found that regardless of TIDM status, Hispanic girls tend to be less physically active than their NHW counterparts [26]. This might partly be attributable to lower socioeconomic status among Hispanic females which has been found to be associated with increased risk for a sedentary lifestyle [24]. 

More frequent weekly physical activity was reported among girls 11–15 years of age with significantly less frequent physical activity reported by girls between 16–19 years of age. Our results are similar to other studies that have also found physical activity to decline and sedentary behavior to become more common during adolescence [27]. Pate et al. specifically examined change in participation in activities during adolescence in girls and found that vigorous physical activity declined from 45.4% in 8th grade to 34.1% in 12th grade. Interestingly, the probability of participating in several forms of vigorous physical activity in 12th grade was strongly associated with participation in those activities in 8th grade [28]. Thus, encouragement of physical activity in those with T1DM at a young age might be important in helping to establish maintenance of physical activity during adolescence and beyond. 

Why are not adolescent females with TIDM more physically active? One study has found that the fear of hypoglycemia was the strongest barrier to regular physical activity [29]. This fear could partly be due to the mismanagement of diabetes during times of exercise. In fact, one study that looked at adolescents with TIDM found that only fifty percent of the patients reported monitoring blood glucose levels during exercise, and only 32% changed insulin dose according to blood glucose levels. Also, hypoglycemic episodes (37.7%) were more frequently reported than hyperglycemic episodes [30]. Thus, the complexities of diabetes surveillance and fear of hypoglycemia during exercise can become a unique challenge to those with TIDM. 

It is also important that health care providers are familiar with ADA guidelines of physical activity. The ADA recommends that blood glucose monitoring should be done before and at the termination of exercise and at hourly intervals during episodes of prolonged strenuous activity. Fifteen grams of carbohydrates may be administered as a readily absorbed sugar if blood glucose levels are <100 mg/dL during the period of exercise [22]. The DirecNet study group has also found that discontinuing basal insulin during exercise is an effective strategy in those using insulin pumps for reducing hypoglycemia [31]. In addition, they have also found that overnight hypoglycemia after exercise is common in children with TIDM and recommend modifying diabetes management following afternoon exercise to reduce the risk of hypoglycemia [32]. Thus, adolescents with TIDM should be involved in comprehensive teaching programs for self-management of diabetes [33] and should be aware that the existing guidelines are useful, but the exact adjustments of insulin dose must be made on an individual basis [34] with the help of their health care provider. 

There were some limitations to our study. First, the participants were predominantly of NHW and Hispanic race/ethnicity, and so we were not able to provide reliable estimates of level of physical activity in other racial/ethnic groups. Also, we did not have a nondiabetic control group to compare physical activity. In addition, there is also the potential for recall bias with the use of self-reported questionnaires. Also, level of energy expenditure is multifactorial and often cannot be well captured by use of a questionnaire [35]. However, similar physical activity questionnaires to the one used in this study have also been found to be valid, reliable, and suitable to use for the purpose of data collection in child and adolescent populations [36]. Further, more frequent reported physical activity was significantly associated with a lower resting pulse and diastolic blood pressure, suggesting that expected physiologic changes were present in association with reported levels of activity.

In conclusion, adolescent females with TIDM report exercising at least 60 minutes a day on 3 days out of a typical week. Only 5% of our subjects met the international recommendations of 60 minutes of moderate-to-vigorous activity per day. Health care providers need to continually encourage adolescent females with T1DM to exercise, and barriers to physical activity need to be reduced, with special attention to Hispanic adolescents and those between 16–19 years of age. Thus, increased physical activity is associated with improved glycemic control in adolescents with TIDM which can ultimately lead to decreased micro- and macrovascular complications of diabetes.

## Figures and Tables

**Table 1:** Characteristics of study participants.

	Total participants (*N* = 203)	Non-Hispanic White (*N* = 172)	Hispanic (*N* = 31)
Age at visit (years)	15.5 ± 2.6	15.5 ± 2.8	15.7 ± 1.8
Duration of diabetes (years)	6.3 ± 4.7	6.4 ± 4.8	6.0 ± 4.1
A1c (%)	9.3 ± 1.9	9.2 ± 1.9	9.8 ± 1.9
Insulin dose (units/kg body weight/day)	0.98 ± 0.34	0.95 ± 0.32	1.1 ± 0.42
BMI (kg/m^2^)	23.3 ± 3.7	23.2 ± 3.5	24.0 ± 4.5
BMI z score	0.41 ± 0.83	0.37 ± 0.81	0.64 ± 0.85
Blood Pressure			
Systolic (mm Hg)	112 ± 14	111 ± 15	114 ± 9
Diastolic (mm Hg)	66 ± 9	66 ± 9	67 ± 9
Resting Pulse	83.5 ± 13.0	83.6 ± 13.1	84.3 ± 12.5
Total Physical activity			
(Number of days over the last week that the subject was physically active for at least 60 minutes per day)	2.6 ± 2.3	2.9 ± 2.3	1.9 ± 2.0∗
Typical Physical activity			
(In a typical week the number of days that they are physically active for at least 60 minutes per day)	3.1 ± 2.2	3.3 ± 2.1	2.3 ± 2.09∗
Met physical activity guidelines (%[12])	4.7%	5.3%	2.5%

*P < .05 for NHW versus Hispanic.

**Table 2:** Level of physical activity by age group.

	Ages 11–15 years (*n* = 97)	Ages 16–19 years (*n* = 116)
Age at visit (years)	13.5 ± 2.7	17.1 ± 1.0^a^
Duration of diabetes (years)	5.0 ± 4.6	7.4 ± 4.4^a^
A1c (%)	9.3 ± 1.9	9.2 ± 1.9
Insulin dose (units/kg body weight/day)	1.04 ± 0.34	0.92 ± 0.34^a^
BMI (kg/m^2^)	0.48 ± 0.84	0.34 ± 0.82
BMI z score		
Blood Pressure		
Systolic (mm Hg)	109 ± 8	114 ± 10^a^
Diastolic (mm Hg)	64 ± 8	68 ± 10
Pulse	84 ± 12.2	83 ± 13.7
Total Physical activity		
(Number of days over the last week that the subject was physically active for at least 60 minutes per day)	3.0 ± 2.2	2.3 ± 2.24^a^
Typical Physical activity		
(In a typical week the number of days that they are physically active for at least 60 minutes per day)	3.6 ± 2.1	2.7 ± 2.2^a^

^a^ *P*-value <.05 compared to 12–15 year age group.

## References

[1] Joslin E (1916). The treatment of diabetes mellitus. *The Canadian Medical Association Journal*.

[2] Lawrence RD (1926). The effect of exercise on insulin action in diabetes. *The British Medical Journal*.

[3] Diabetes Control and Complications Research Group (1993). The effect of intensive treatment of diabetes on the development and progression of long-term complications in insulin-dependent diabetes mellitus. *New England Journal of Medicine*.

[4] Austin A, Warty V, Janosky J, Arslanian S (1993). The relationship of physical fitness to lipid and lipoprotein(a) levels in adolescents with IDDM. *Diabetes Care*.

[5] Rasmussen OW, Lauszus FF, Hermansen K (1994). Effects of postprandial exercise on glycemic response in IDDM subjects: studies at constant insulinemia. *Diabetes Care*.

[6] Corigliano G, Iazzetta N, Corigliano M, Strollo F (2006). Blood glucose changes in diabetic children and adolescents engaged in most common sports activities. *Acta Biomedica de l’Ateneo Parmense*.

[7] Heyman E, Briard D, Gratas-Delamarche A, Delamarche P, De Kerdanet M (2005). Normal physical working capacity in prepubertal children with type 1 diabetes compared with healthy controls. *Acta Paediatrica*.

[8] Blair SN, Cheng Y, Holder JS (2001). Is physical activity or physical fitness more important in defining health benefits?. *Medicine and Science in Sports and Exercise*.

[9] Penedo FJ, Dahn JR (2005). Exercise and well-being: a review of mental and physical health benefits associated with physical activity. *Current Opinion in Psychiatry*.

[10] Wiesinger GF, Pleiner J, Quittan M (2001). Health related quality of life in patients with long-standing insulin dependent (type 1) diabetes mellitus: benefits of regular physical training. *Wiener Klinische Wochenschrift*.

[11] Stephens T (1988). Physical activity and mental health in the United States and Canada: evidence from four population surveys. *Preventive Medicine*.

[12] Crews DJ, Lochbaum MR, Landers DM (2004). Aerobic physical activity effects on psychological well-being in low-income hispanic children. *Perceptual and Motor Skills*.

[13] Herbst A, Bachran R, Kapellen T, Holl RW (2006). Effects of regular physical activity on control of glycemia in pediatric patients with type 1 diabetes mellitus. *Archives of Pediatrics and Adolescent Medicine*.

[14] Ligtenberg PC, Blans M, Hoekstra JBL, Van Der Tweel I, Erkelens DW (1999). No effect of long-term physical activity on the glycemic control in type 1 diabetes patients: a cross-sectional study. *Netherlands Journal of Medicine*.

[15] Åman J, Skinner TC, de Beaufort CE (2009). Associations between physical activity, sedentary behavior, and glycemic control in a large cohort of adolescents with type 1 diabetes: the Hvidoere Study Group on Childhood Diabetes. *Pediatric Diabetes*.

[16] Särnbladn S, Ekelund U, Åman J (2005). Physical activity and energy intake in adolescent girls with type 1 diabetes. *Diabetic Medicine*.

[17] Øverby NC, Margeirsdottir HD, Brunborg C, Anderssen SA, Andersen LF, Dahl-Jørgensen K (2009). Physical activity and overweight in children and adolescents using intensified insulin treatment. *Pediatric Diabetes*.

[18] Domargård A, Särnblad S, Kroon M, Karlsson I, Skeppner G, Åman J (1999). Increased prevalence of overweight in adolescent girls with type 1 diabetes mellitus. *Acta Paediatrica*.

[19] American Diabetes Association (2005). Diagnosis and classification of diabetes. *Diabetes Care*.

[20] Prochaska JJ, Sallis JF, Long B (2001). A physical activity screening measure for use with adolescents in primary care. *Archives of Pediatrics and Adolescent Medicine*.

[21] Strong WB, Malina RM, Blimkie CJR (2005). Evidence based physical activity for school-age youth. *Journal of Pediatrics*.

[22] Silverstein J, Klingensmith G, Copeland K (2005). Care of children and adolescents with type 1 diabetes: a statement of the American Diabetes Association. *Diabetes Care*.

[23] Council on Sports Medicine and Fitness (2006). Active healthy living: prevention of childhood obesity through increased physical activity. *Pediatrics*.

[24] Singh GK, Kogan MD, Van Dyck PC, Siahpush M (2008). Racial/ethnic, socioeconomic, and behavioral determinants of childhood and adolescent obesity in the United States: analyzing independent and joint associations. *Annals of Epidemiology*.

[25] Lachin JM, Genuth S, Nathan DM, Zinman B, Rutledge BN (2008). Effect of glycemic exposure on the risk of microvascular complications in the diabetes control and complications trial-revisited. *Diabetes*.

[26] Pate RR, Stevens J, Webber LS (2009). Age-related change in physical activity in adolescent girls. *Journal of Adolescent Health*.

[27] Brodersen NH, Steptoe A, Boniface DR, Wardle J (2007). Trends in physical activity and sedentary behaviour in adolescence: ethnic and socioeconomic differences. *British Journal of Sports Medicine*.

[28] Pate RR, Dowda M, O’Neill JR, Ward DS (2007). Change in physical activity participation among adolescent girls from 8th to 12th grade. *Journal of Physical Activity & Health*.

[29] Brazeau A-S, Rabasa-Lhoret R, Strychar I, Mircescu H (2008). Barriers to physical activity among patients with type 1 diabetes. *Diabetes Care*.

[30] Bernardini AL, Vanelli M, Chiari G (2004). Adherence to physical activity in young people with type 1 diabetes. *Acta Biomedica de l’Ateneo Parmense*.

[31] The Diabetes Research in Children Network (DirecNet) Study Group (2006). Prevention of hypoglycemia during exercise in children with type 1 diabetes by suspending basal insulin. *Diabetes Care*.

[32] The Diabetes Research in Children Network (DirecNet) Study Group (2005). Impact of exercise on overnight glycemic control in children with type 1 diabetes mellitus. *Journal of Pediatrics*.

[33] Kemmer FW (1992). Prevention of hypoglycemia during exercise in type I diabetes. *Diabetes Care*.

[34] Toni S, Reali MF, Barni F, Lenzi L, Festini F (2006). Managing insulin therapy during exercise in type 1 diabetes mellitus. *Acta Biomedica de l’Ateneo Parmense*.

[35] Wareham NJ (2001). Commentary: measuring physical activity in Sub-Saharan Africa. *International Journal of Epidemiology*.

[36] Wong SL, Leatherdale ST, Manske S (2006). Reliability and validity of a school-based physical activity questionnaire. *Medicine and Science in Sports and Exercise*.

